# Towards MRI-Only Mandibular Resection Planning: CT-like Bone Segmentation from Routine T1 MRI Images Using Deep Learning

**DOI:** 10.3390/cmtr18030040

**Published:** 2025-09-19

**Authors:** Reinier S. A. ten Brink, Bram J. Merema, Marith E. den Otter, Willemina A. van Veldhuizen, Max J. H. Witjes, Joep Kraeima

**Affiliations:** 1 Department of Maxillofacial Surgery, University Medical Center Groningen, 9713GZ Groningen, The Netherlands; b.j.merema@umcg.nl (B.J.M.); m.j.h.witjes@umcg.nl (M.J.H.W.); j.kraeima@umcg.nl (J.K.); 2 3D Lab, University Medical Center Groningen, 9713GZ Groningen, The Netherlands; marithdenotter@outlook.com (M.E.d.O.); w.a.van.veldhuizen@umcg.nl (W.A.v.V.)

**Keywords:** deep learning, magnetic resonance imaging, surgery, computer-assisted, surgery, oral

## Abstract

We present a deep learning-based approach for accurate bone segmentation directly from routine T1-weighted MRI scans, with the goal of enabling MRI-only virtual surgical planning in head and neck oncology. Current workflows rely on CT for bone modeling and MRI for tumor delineation, introducing challenges related to image registration, radiation exposure, and resource use. To address this, we trained a deep neural network using CT-based segmentations of the mandible, cranium, and inferior alveolar nerve as ground truth. A dataset of 100 patients with paired CT and MRI scans was collected. MRI scans were resampled to the voxel size of CT, and corresponding CT segmentations were rigidly aligned to MRI. The model was trained on 80 cases and evaluated on 20 cases using Dice similarity coefficient, Intersection over Union (IoU), precision, and recall. The network achieved a mean Dice of 0.86 (SD ± 0.03), IoU of 0.76 (SD ± 0.05), and both precision and recall of 0.86 (SD ± 0.05). Surface deviation analysis between CT- and MRI-derived bone models showed a median deviation of 0.21 mm (IQR 0.05) for the mandible and 0.30 mm (IQR 0.05) for the cranium. These results demonstrate that accurate CT-like bone models can be derived from standard MRI, supporting the feasibility of MRI-only surgical planning.

## 1. Introduction

3D virtual surgical planning (VSP) is applied in the current standard of care for head and neck oncological surgery for more than a decade already [1,2,3]. This technology enables surgeons to preoperatively visualize complex anatomy, plan surgeries preoperatively, and design 3D printed surgical guides and patient-specific implants (PSIs). This workflow requires rigid matching between MRI and CT, to obtain both soft tissue, tumour and bone tissue delineation, which introduces a 1–2 mm error due to anatomical changes between scans [4]. To mitigate the clinical consequences of these errors misalignments, safety margins are added around the tumour, resulting in unnecessarily larger bone resections.

Furthermore, the requirement for multiple imaging modalities places additional burdens on patients, including increased time and radiation exposure from CT scans. Additionally, the need for extra scans causes strain on hospital resources, contributing to longer waiting times, higher healthcare costs and energy consumption [5].

Given these challenges, a single-modality VSP workflow could significantly reduce the required number of scans needed and remove the error introduced by MRI-CT fusion. MRI, which is better for tumour delineation, is the preferred modality, but accurate bone models from MRI are essential [6,7]. Previous studies have explored the use of blackbone MRI sequences for bone segmentation as a potential solution [8,9,10,11,12,13]. However, this method adds an additional scan to the already long MRI protocol and requires extensive laborious manual adjustments to accurately segment, limiting clinical feasibility.

This retrospective study investigates whether T1 VIBE Dixon MRI scans, already part of our routine imaging protocol, can be leveraged for bone segmentation. We propose training a deep learning model on MRI scans that uses CT-based bone segmentations as ground truth labels to enable accurate and efficient bone segmentation from these MRI scans. This would lay the foundation for a fully MRI-based VSP workflow that reduces the reliance on CT, minimizes registration errors, and optimizes the overall surgical planning process.

## 2. Materials and Methods

### 2.1. Dataset

The retrospective dataset consisted of N = 100 paired T1 MRI scans and CT scans acquired between 2018 and 2025 during routine diagnostic work-up of oncology patients from the department of Oral and Maxillofacial Surgery of the University Medical Center Groningen (UMCG), The Netherlands. Approval for this study was obtained from the Central Ethics Committee of the University Medical Center Groningen (CTc 188984). The included patients were diagnosed with a tumour in the oral cavity region with a planned mandibulectomy or maxillectomy. Patients with dental implants in their jaw were excluded since this causes major artifacts in the MRI. Due to anonymization protocols and the random selection of scans, detailed demographic or pathological information was not available.

The CT scans were acquired in headfirst supine position with either a Siemens SOMATOM Definition AS scanner, or a Siemens SOMATOM Force scanner (Siemens Healthineers, Forchheim, Germany). The resulting CT scans had a median voxel size of 0.6 × 0.6 × 0.6. The MRI scans were acquired in headfirst supine position with a 3.0 Tesla MRI scanner (SIEMENS Magnetom Prisma, Siemens Healthineers, Forchheim, Germany), with a 64-channel head coil. The scans were obtained with the suprahyoid protocol in the T1-weigthed gradient echo (Dixon VIBE) sequence with contrast agent DOTAREM. In-phase scans were used within this study. The parameters of the MRI were: repetition time/echo time, 5.5/2.46 ms; matrix, 256 × 256 and voxel size 0.9 × 0.9 × 0.9 mm. In order to ensure that the bone models from the CT would fit to the MRI, the MRI scans were resampled to 0.6 × 0.6 × 0.6 mm using linear interpolation and then transformed into nii.gz files.

Manual segmentation of the CT scans was performed using Mimics 26.0 research software (Materialise, Leuven, Belgium). The mandible, cranium and bilateral inferior alveolar nerve (NAI) were segmented. For the thin orbital bone, Mimics’ thin bone segment tool was utilized. For the NAI, a spline of 2 mm diameter was drawn through the mandibular canal from the mandibular foramen to the mental foramen. The manual segmentations were performed by a technical physician with 4 years of experience. After creating the 3D models from the segmentations, all segmentations were exported to a standard tessellation language (STL) format and imported into the 3-Matic 18.0 research software (Materialise, Leuven, Belgium). In this software all bones were wrapped with a gap closing distance of 3.0 mm, getting rid of any holes smaller than 3.0 mm, and a detail level of 0.5 mm. These parameters were determined empirically. The resulting models were exported as STL files.

**Figure 1:**
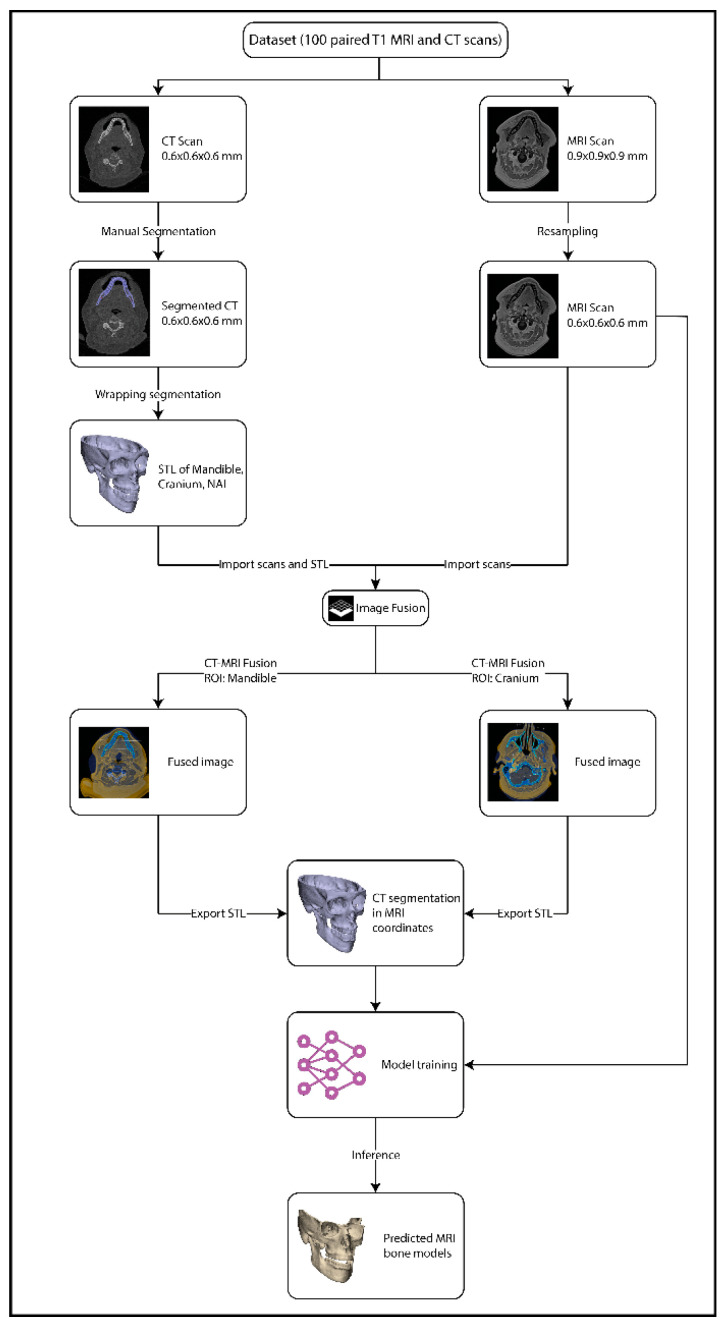
The image processing workflow.

All STL files were uploaded to Brainlab software 4.5 (Brainlab, München, Germany) and linked to the corresponding CT scan. The image fusion tool was then used to align the CT scan with the T1 MRI scan. Since neither the CT or the MRI was acquired in occlusion, this process was repeated twice, to overcome the positional difference in the bones between the MRI and CT, by an experienced operator (RtB): once with the region of interest (ROI) set on the mandible during fusion, after which the mandible and NAI STL files were exported; and once with the ROI set on the cranium, after which the cranium STL file was exported. These STL files were transformed into nii.gz files to serve as a mask in training. The image processing workflow is illustrated in Figure 1.

### 2.2. AI Framework

The nnU-Net convolutional neural network was selected as the framework for automatic segmentation, given its versatility across different datasets [14]. The model was implemented on an Intel^®^ Core™ i9-10980XE CPU with 64 GB RAM and a graphic card of NVIDIA RTX A4000 (NVIDIA Corporation, Santa Clara, CA, USA) with 16 GB GDDR6 memory. A split of 80% for training and 20% for testing was used. Training was carried out using the 3D full resolution configuration with 5-fold cross validation and 1000 epochs per fold using the cross-entropy dice loss function using a polynomial learning rate scheduler with initial learning rate of 0.01. Following the training process, segmentation predictions on the test set were generated by averaging the SoftMax outputs of all 5 trained folds and selecting the label with the highest probability per voxel to achieve higher accuracy and reliability.

### 2.3. Evaluation Metrics

Inference on the MRI scans in the test set produced the MRI-based segmentations which were compared against the CT-derived labels rigidly aligned to MRI. The following classification metrics were used to evaluate the performance of the model on the test dataset:

$$\mathrm{Dice}:DSC=\frac{2\left|A\cap B\right|}{\left|A\right|+\left|B\right|}$$

$$\mathrm{Intersection}\;\mathrm{over}\;\mathrm{Union}:\mathrm{IoU}=\frac{\left|A\cap B\right|}{\left|A\right|\cup \left|B\right|}$$

$$\mathrm{Precision}:Precision=\frac{True\;positives}{True\;positives+False\;positives}$$

$$\mathrm{Recall}:Recall=\frac{True\;positives}{True\;positives+False\;negatives}$$

$$95\mathrm{th}\;\mathrm{percentile}\;\mathrm{Hausdorff}\;\mathrm{distance}:H(A,B)=max\;\{h_{95}(A,B),h_{95}(B,A)\}$$

The MRI derived bone models were matched to the original CT bone model using the global registration tool in 3-Matic. To compare the CT and MRI-based bone models, median deviation was calculated and distance maps generated using part-to-part comparison in 3-Matic. The analysis was restricted to bone regions that were present in both the CT and MRI scans to ensure a consistent comparison. A median deviation of ≤1 mm between the CT and MRI was deemed acceptable for clinical use based on clinical consensus.

## 3. Results

**Figure 2:**
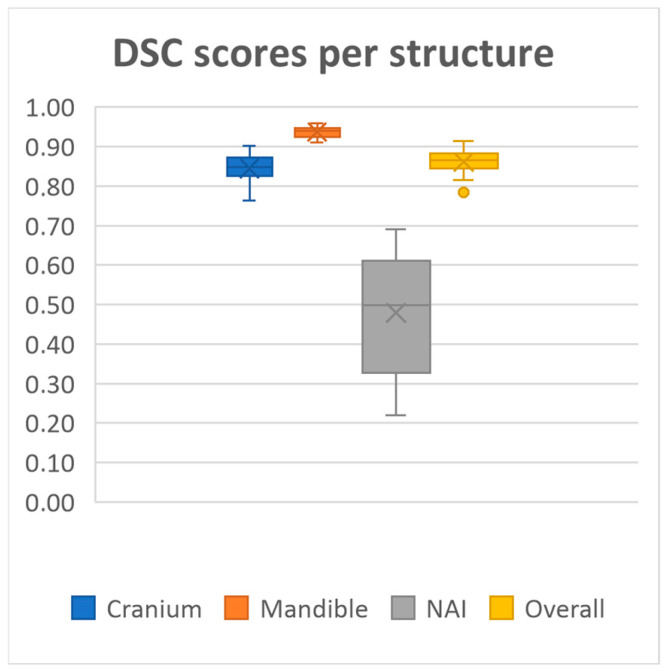
Median Dice scores per structure and all structures combined.

**Figure 3:**
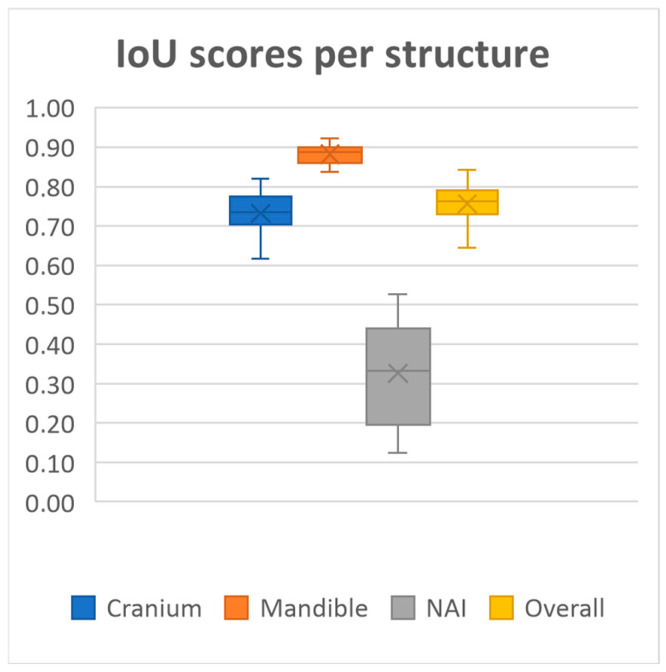
Median Intersection over Union scores per structure and all structures combined.

**Figure 4:**
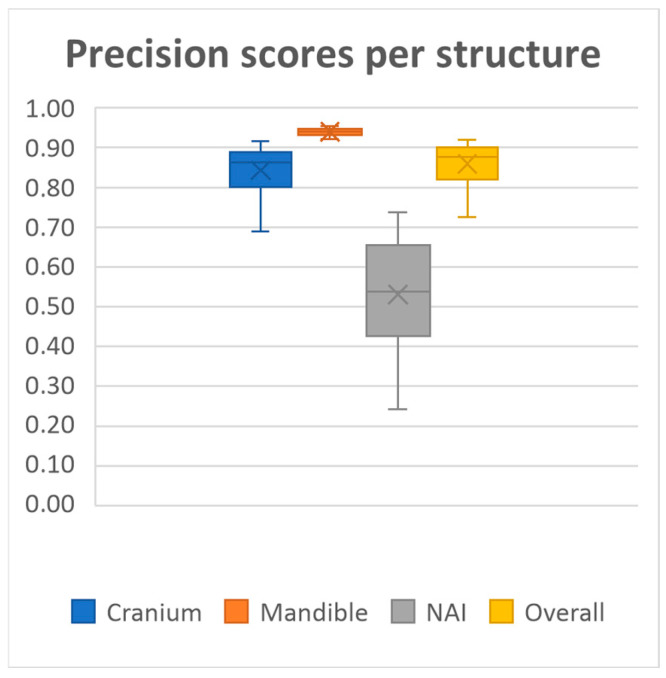
Median precision scores per structure and all structures combined.

**Figure 5:**
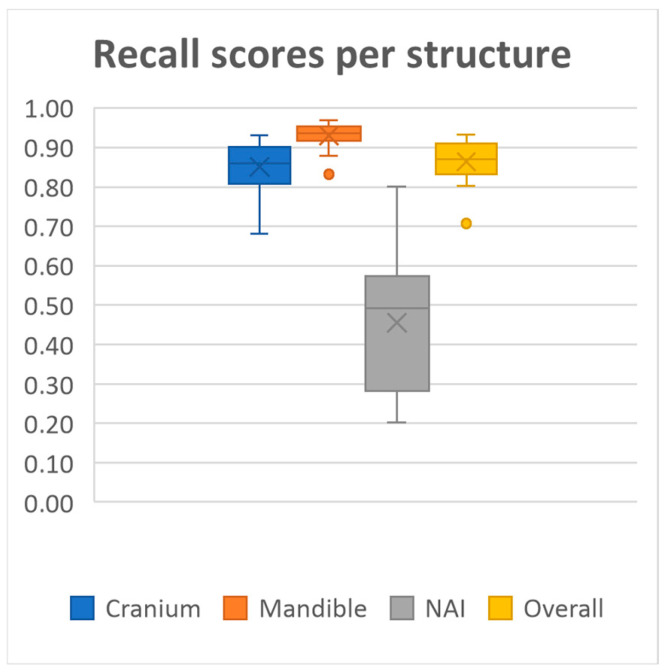
Median recall scores per structure and all structures combined.

20 MRI-based mandible and cranium models were obtained using our deep learning model. The model achieved overall mean Dice similarity coefficient of 0.86 (SD ± 0.03, range 0.78–0.91), Intersection over Union of 0.76 (SD ± 0.05, range 0.64–0.84), precision of 0.86 (SD ± 0.05, range 0.72–0.92) and recall of 0.86 (SD ± 0.05, range 0.71–0.93) on the test dataset. A summary of the evaluation metrics for each anatomical structure on the test dataset is presented in Figure 2, Figure 3, Figure 4 and Figure 5 and Table 1.

Performance was consistently high for both the mandible and cranium, with the mandible achieving the highest scores, as well as the lowest standard deviations across all metrics (Dice 0.94 ± 0.01, IoU, 0.88 ± 0.02, Precision 0.94 ± 0.01, Recall 0.93 ± 0.03, HD95 1.60 ± 0.42). The cranium segmentation performed slightly lower across all metrics, indicating a more variable segmentation performance compared to the mandible (Dice 0.84 ± 0.03, IoU, 0.73 ± 0.05, Precision 0.84 ± 0.06, Recall 0.85 ± 0.06, HD95 4.71 ± 2.07). In contrast, segmentation performance for the NAI was considerably lower than those of the bony structures, highlighting difficulty in accurately segmenting the NAI (Dice 0.48 ± 0.14, IoU, 0.33 ± 0.12, Precision 0.53 ± 0.14, Recall 0.46 ± 0.16, HD95 2.77 ± 1.28).

**Figure 6:**
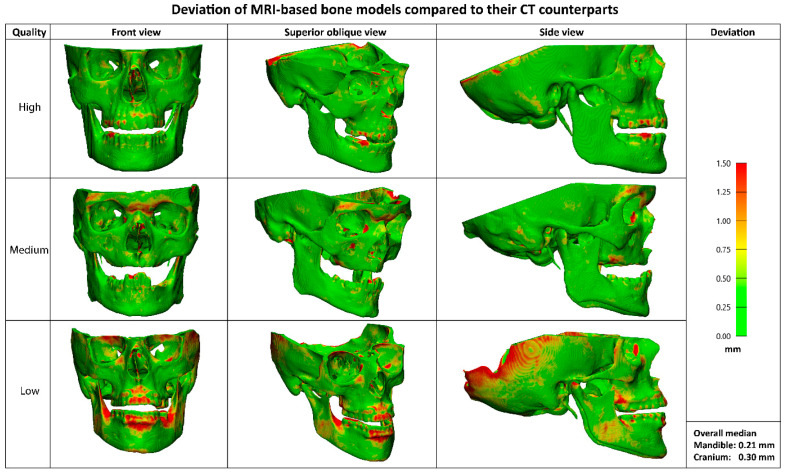
Visualisation of MRI-based bone models for a high-, medium- and low-quality result. The distance map shows deviations compared to their CT counterparts, with green representing small differences (0–0.5 mm) and red representing large differences (>1 mm). Localized larger deviations are qualitatively observed in the dorsal part of the cranium, mandibular condyles, and teeth.

The median deviation between the CT and MRI-based bone models was 0.21 mm (IQR 0.05) for the mandible and 0.30 mm (IQR 0.08) for the cranium. Visual representations of the MRI-based bone models in Figure 6 illustrates the deviations compared to their CT counterparts. While no separate segmentations of sub anatomy were evaluated, localized larger deviations (>1mm) were qualitatively observed in the dorsal part of the cranium, mandibular condyles, and teeth.

## 4. Discussion

This study shows that cranium and mandibula models can be segmented accurately and fast from T1 MRI scans with comparable results as CT-based segmentation of bony tissue and without manually laborious work. This is a significant step towards fully MRI-based VSPs, surgical guide design and incorporating soft tissue into resection and reconstruction planning.

While previous studies have shown that accurate bone segmentation from MRI is possible, it typically involves many laborious steps, such as extensive manual annotation or post-processing due to the lack of a unique intensity range for bone in MRI and resulting in increased risk of variability between observers [7,12]. These approaches have also relied on dedicated MRI sequences, such as Black Bone VIBE, which add to the imaging protocol and may not be widely available or feasible for all patient groups [8,9,10,11,12,13]. These factors have limited the adoption of MRI-only workflows in 3D VSP. This study preserves the advantages of MRI-only planning and eliminates the need for manual segmentation or specialized sequences, thereby significantly reducing the time and expertise required for bone model generation and making MRI-only workflows accessible and efficient.

The ability to generate accurate bone models from MRI is particularly valuable for designing patient-specific implants and surgical guides for tumour resection. Several studies have explored deep learning-based bone segmentation from MRI, often relying on manual annotations as ground truth or requiring modifications to scanning protocols [11,15,16]. Our method circumvents these limitations by leveraging paired CT and MRI data for deep learning model training, producing bone models form MRI only that are comparable to CT-based reconstructions. These findings could potentially simplify imaging protocols for head and neck cancer patients. This would eliminate the need for MRI-CT matching, creating a more accurate VSP which might reduce the necessary tumour margin and therefore bone resections.

By eliminating the need for a dedicated head CT, radiation exposure to sensitive structures such as the cornea and thyroid gland could be significantly reduced. Reducing the number of necessary CT scans might decrease the strain on hospital resources. Our approach could also be extended to other anatomical regions, provided paired datasets are available.

One important consideration might be the continued necessity of thoracic CT for assessing pulmonary nodules and metastases in oncological cases. Additionally, differences in spatial resolution between CT and MRI may impact the radiological evaluation of subtle bone invasions. To address these challenges, a triage system could be implemented to determine when both CT and MRI are necessary versus when MRI alone is sufficient.

The most significant deviations (>1 mm) between the CT and MRI-derived bone models occur in the dorsal part of the cranium, the mandibular condyles, and the teeth. The missing part of the cranium is likely due to differences in scan coverage between the MRI and CT in the training dataset. It could potentially be improved by manually adding this segmentation to the MRI or building a dataset where the CT fully covers the same area as the MRI. This also explains the higher Hausdorff scores of the cranium. Deviation in the condyles could be explained by the fact that it is difficult to differentiate the condyle from the glenoid fossa due to the articular disc cartilage that is picked up on MRI. [17] The deviation in the teeth can be explained by several aspects. First, the segmentation of the teeth from the CT contains artefacts due to the presence of tooth fillings. Moreover, teeth are relatively small and the original MRI scan has a voxel size of 0.9 mm, thus not capturing the entire tooth. Resampling the scan does not add this data to the scan. All three of these deviations do not affect the clinical usability of the model, as all areas are not essential for designing surgical guides or patient-specific implants.

The accuracy of nerve segmentation remains a challenge. Automatic segmentation of the NAI from MRI is not yet clinically usable with this model (Dice 0.48 ± 0.14). This may be due to the discrepancy between the structures segmented on CT versus MRI. On CT, the mandibular canal is segmented as a proxy for the nerve, while MRI allows direct visualization of the nerve itself, which is smaller than the canal. This mismatch may have impacted segmentation performance. Given the importance of precise NAI modelling for patient-specific implant design, future work should explore whether increasing the dataset size or manually annotating the NAI on MRI could improve segmentation accuracy. However, manual annotation of the NAI on MRI is a relatively easy and fast task, therefore this does not limit clinical usability of the model.

To fully transition to an MRI-based 3D mandibular reconstruction workflow, segmentation of the fibula from MRI scans is required. Future work should focus on acquiring paired CT and MRI scans of the lower leg to develop a segmentation model for fibular graft planning, currently a study protocol and method is developed in our hospital. A prospective study where an MRI-only workflow will be implemented is needed to test the feasibility of our method in clinical practice.

## 5. Conclusions

In conclusion, this study shows that a deep learning model, trained on 3D CT reconstructions as input labels, can accurately and fast segment the cranium and mandible from routine T1 MRI scans only, resulting in very accurate bone models. This paves the way for fully MRI-based virtual surgical planning, incorporating soft tissue planning and reducing reliance on CT scans in head and neck cancer treatment.

## 6. Patents

The authors declare that a patent application related to aspects of this study has been filed but not yet granted.

## Figures and Tables

**Table 1:** Mean evaluation metrics of the model performance on the test set.

	Structure	Mandible	Cranium	Inferior Alveolar Nerve (NAI)	Overall
Metric					
Dice (SD)		0.94 (±0.01)	0.84 (±0.03)	0.48 (±0.14)	0.86 (±0.03)
IoU (SD)		0.88 (±0.02)	0.73 (±0.05)	0.33 (±0.12)	0.76 (±0.05)
Precision (SD)		0.94 (±0.01)	0.84 (±0.06)	0.53 (±0.14)	0.86 (±0.05)
Recall (SD)		0.93 (±0.03)	0.85 (±0.06)	0.46 (±0.16)	0.86 (±0.05)
95th Hausdorff (HD)		1.60 (±0.42)	4.71 (±2.07)	2.77 (±1.28)	-

## Data Availability

The data supporting the conclusions of this article will be made available by the authors on reasonable request.

## Abbreviations

The following abbreviations are used in this manuscript:

CT	Computed Tomography
HD	Hausdorff Distance
IoU	Intersection over Union
IQR	Interquartile Range
MRI	Magnetic Resonance Imaging
NAI	Nervus Alveolaris Inferior
ROI	Region of Interest
SD	Standard Deviation
STL	Standard Tesselation Language
UMCG	University Medical Center Groningen
VSP	Virtual Surgical Planning
